# Treatment effect prediction with adversarial deep learning using electronic health records

**DOI:** 10.1186/s12911-020-01151-9

**Published:** 2020-12-14

**Authors:** Jiebin Chu, Wei Dong, Jinliang Wang, Kunlun He, Zhengxing Huang

**Affiliations:** 1 College of Biomedical Engineering and Instrumental Science, Zhejiang University, Hangzhou, China; 2 Department of Cardiology, Chinese PLA General Hospital, Beijing, China; 3 Cardiocloud Medical Technology, Beijing, China

**Keywords:** Treatment effect prediction, Deep learning, Adversarial learning, Electronic health records

## Abstract

**Background:**

Treatment effect prediction (TEP) plays an important role in disease management by ensuring that the expected clinical outcomes are obtained after performing specialized and sophisticated treatments on patients given their personalized clinical status. In recent years, the wide adoption of electronic health records (EHRs) has provided a comprehensive data source for intelligent clinical applications including the TEP investigated in this study.

**Method:**

We examined the problem of using a large volume of heterogeneous EHR data to predict treatment effects and developed an adversarial deep treatment effect prediction model to address the problem. Our model employed two auto-encoders for learning the representative and discriminative features of both patient characteristics and treatments from EHR data. The discriminative power of the learned features was further enhanced by decoding the correlational information between the patient characteristics and subsequent treatments by means of a generated adversarial learning strategy. Thereafter, a logistic regression layer was appended on the top of the resulting feature representation layer for TEP.

**Result:**

The proposed model was evaluated on two real clinical datasets collected from the cardiology department of a Chinese hospital. In particular, on acute coronary syndrome (ACS) dataset, the proposed adversarial deep treatment effect prediction (ADTEP) (0.662) exhibited 1.4, 2.2, and 6.3% performance gains in terms of the area under the ROC curve (AUC) over deep treatment effect prediction (DTEP) (0.653), logistic regression (LR) (0.648), and support vector machine (SVM) (0.621), respectively. As for heart failure (HF) case study, the proposed ADTEP also outperformed all benchmarks. The experimental results demonstrated that our proposed model achieved competitive performance compared to state-of-the-art models in tackling the TEP problem.

**Conclusion:**

In this work, we propose a novel model to address the TEP problem by utilizing a large volume of observational data from EHR. With adversarial learning strategy, our proposed model can further explore the correlational information between patient statuses and treatments to extract more robust and discriminative representation of patient samples from their EHR data. Such representation finally benefits the model on TEP. The experimental results of two case studies demonstrate the superiority of our proposed method compared to state-of-the-art methods.

## Background

Defined as the operations and medication delivered during hospitalization, treatments have a significant impact on the prognosis of patients. Are the treatment interventions appropriate to be conducted on an individual patient given his or her specific clinical status? Will the delivered treatments achieve the expected effects on patients during their hospitalization? Traditional approaches to addressing such questions have mostly relied on evidence-based medicine [1], which urges healthcare professionals to make treatment decisions according to the best evidence from systematic research on both the efficacy and efficiency of various therapeutic alternatives [2]. Ideally, healthcare professionals compare different treatment options by referring to randomized, double-blind, head-to-head clinical trials [1], evaluate the resulting treatment effects in a prospective manner, and then select the best one to be conducted on individuals according to their specific clinical status [3].

Although valuable, there are two typical limitations to randomized controlled trial (RCT) studies [1, 4–8]. The first is that participants in RCTs are strictly selected and tend to be a “pretty rarefied population”, which is not representative of the real-world population that the scheduled treatments will eventually target [5, 6]. The second is that existing approaches are almost from a reactive perspective, in that they allow healthcare professionals to identify inappropriate interventions only after they have occurred, rather than supporting them in preventing unexpected treatment effects in advance [7, 8].

Electronic health records (EHRs), with their increasingly widespread adoption in clinical practice, provide a comprehensive source for treatment effect analysis to augment traditional RCT studies [9–15]. An EHR contains large amounts of clinical data generated as a byproduct of treatment activities [10]. A wide variety of data types are available in EHRs, including patient demographics, symptoms, vital signs, laboratory test results, and other data types that can be used to describe a patient’s clinical status, and therefore subsequent treatments (for example, drugs, injections, surgery, and care activities) performed on the patient conditioned on his or her clinical status [14, 15]. In this regard, the different aspects of medical information recorded in EHR data are highly correlated and thus provide significant potential for exploitation, for example, to extract representative and discriminative features for treatment effect prediction (TEP), which is the main objective of this study.

TEP is vital for efficiently managing disease care and therapy, owing to its usefulness in capturing actionable knowledge to assist healthcare professionals in selecting among the many therapies claimed to be efficacious for treating a patient within a specific clinical status [16–19]. As a fundamental problem of precision medicine with a wide range of applications, such as treatment recommendation [20, 21] and medical error avoidance [22], TEP can generate non-trivial knowledge with dual benefits. Not only can it demonstrate comprehension regarding patient treatment adoption, but it can also serve as an efficient and proactive indicator of medical errors before they actually occur.

To address the challenges of TEP, EHR-driven models are generally required to be capable of capturing representative and discriminative features of patient characteristics and subsequent treatments in an integrated manner and from a large volume of EHR data. In this study, we use deep learning tactics to leverage the potential of EHR data to anticipate treatment effects. Specifically, we propose a novel adversarial deep learning model for treatment effect prediction (ADTEP) based on the auto-encoder (AE) [23, 24] and adversarial learning [25]. In detail, we employ two AEs, which encode the physical condition and treatment information of patient samples into latent robust representations. In addition to the treatment decoder, treatments can be generated based on the latent representation of the patient status, under the manipulation of the actual treatment effect, so as to regularize the latent features and capture correlations between patient characteristics and treatments. To align the generated treatments with the actual performed treatments, we adopt an adversarial learning scheme and use a discriminator to differentiate the fake generated treatments from the real performed treatments documented in the EHR data. With this adversarial learning strategy, not only the patient characteristics and subsequent treatments, but also the correlational information between them are encoded in the latent representation, making the generated features sufficiently representative to convey the essential and critical information in the EHR data. Note that the latent representations of patient samples and the treatment effect predictor are jointly trained, making the representations discriminative and optimized for TEP. We conducted experiments to evaluate the effectiveness of the proposed model on two real clinical data sets collected from the Cardiology Department of the Chinese PLA General hospital. The experimental results demonstrate that our proposed model outperforms other state-of-the-art models.

The remainder of this paper is organized as follows. We review the related work in Section 2. Section 3 formulates the problem and presents our proposed approach in detail. The experimental setup and results using a real clinical dataset are presented in Section 4. Finally, we conclude the paper in Section 5.

## Related work

TEP models [16–18, 26–33] have been proposed to predict the treatment effects of patient individuals following the performed treatment. The gold standard approach to addressing the problem of TEP is clinical RCTs, which aim to avoid bias when testing new treatments [5]. Although valuable, RCTs exhibit several serious limitations [6–8]; for example, they require strict inclusion and exclusion criteria, the causal conclusions from RCT studies cannot be applied to other localities automatically, and most importantly, RCTs are sometimes infeasible owing to ethical issues.

In recent years, the increased availability of EHRs has demonstrated significant potential for improving the performance of various clinical applications [9–15, 34, 35]. EHRs regularly document various care and treatment behaviors, such as procedures, diagnoses, and laboratory tests and measurements, of patients within the context of large healthcare systems [9, 10], capture the characteristics of heterogeneous populations of patients receiving care in their current clinical setting [11], and therefore form a large volume of clinical observational data sources. As an essential source of clinical observational data, and an efficient and alternative channel for TEP, EHR data have been gradually incorporated into estimating treatment effects [36, 37]. For example, Rosenbaum and Rubin proposed a classical propensity score matching model to reduce selection bias for estimating treatment effects [2]. Wager and Athey [26] proposed a variant of random forests, known as causal forests, to measure the propensity scores for treatment effect estimation.

Although valuable, two main limitations exist when using EHR data for TEP: (1) treatment selection bias inevitably exists in clinical practice [16, 17], that is, similar patients always receive the same treatments based on the recommendations from certain pre-existing clinical guidelines or protocols, and thus, EHR data are typically biased as they faithfully documents the actual treatment behavior and do not contain all possible outcomes for all treatments; (2) only the factual outcomes of the assigned treatments are observed, and counterfactual outcomes of alternative treatments are not observed [26–29, 33, 36, 37]. Note that the treatment outcomes of patients are never the same, and therefore, the learning process must provide an understanding of how the current patient is similar to previous patients [30]. This learning problem is further complicated by the fact that the data include only the received treatment outcomes, and - not the potential outcomes of the alternative treatments, namely the counterfactuals [27].

To overcome these limitations, numerous studies have proposed creating a balance by re-weighting samples with their inverse propensity score (IPS) and formulating the problem of counterfactual inference as the domain adaption problem [28, 29]. For example, Swaminathan and Joachims proposed a direct estimation model to minimize the “corrected” loss function, using IPS corrected by a regularization term over the linear stochastic policy class [28]. As a further study, Swaminathan and Joachims developed a variant of the IPS estimator, that is, a self-normalizing estimator, to learn the counterfactuals [30]. Jordan and Schaar proposed combining the direct and IPS methods and generate more robust counterfactual estimates [30]. In particular, they used a novel AE network to reduce bias by learning a representation map to control the trade-off between the bias reduction and information loss [30].

In recent years, deep learning has attracted considerable interest in various research fields for achieving impressive performance. Shifting to the clinical domain, deep learning tactics have been receiving increased attention for solving the TEP problem [17, 27, 30, 32]. For example, Louizos et al. [16] proposed the causal effect variational AE to learn the latent variables for estimating individual treatment effects. Atan, Jordan and Schaar [30] proposed a deep-treat model to estimate the treatment policies on the transformed data learned from an AE. Lee et al. [31] developed a novel adversarial learning framework to conduct unbiased treatment effect estimation using noisy proxies. Yoon et al. [17] employed a generative adversarial network (GAN) to estimate individual treatment effects. Alaa et al. [33] proposed multi-task deep counterfactual networks for treatment effect estimation by learning shared representations for treated and control outcomes and reducing the impact of selection bias in observational data by means of a propensity-dropout regularization scheme. Although valuable, it must be mentioned that most of these deep learning models have assumed that only binary actions or a few treatment options exist, namely treat and do not treat, while in most situations, various treatment combinations are possible.

In comparison with state-of-the-art models that simply tackle binary or several treatment options, our proposed ADTEP elegantly deals with various treatment combinations by extracting representative and discriminative features from observational data. Moreover, the proposed model is capable of extracting correlational information between patient characteristics and treatments from EHR data, which is essential for treatment effect estimation but somehow neglected by numerous existing models.

## Methods

(***x***: patient feature vector, *y*: outcome, ***a***: treatment vector, ***h***_*x*_: latent feature vector of patient features, ***h***_*a*_: latent feature vector of treatments, ***x***^′^**:** reconstructed feature vector of patient features, ***a***^′^: reconstructed feature vector of treatments, $\overset{\sim }{\boldsymbol{a}}$: fabricated vector of treatments, E_x_: patient feature encoder, E_a_: treatment intervention encoder, G_x_: patient feature decoder, G_a_: treatment decoder, G_xa_: treatment generator, D_a_: treatment discriminator, C_y_: logistic regression layer for TEP, *l*_*x*_: patient feature reconstruction loss, *l*_*a*_: treatment reconstruction loss, *l*_*GAN*_: adversarial loss, *l*_*pred*_: treatment outcome prediction loss.)

We consider a typical clinical study of TEP, in which the EHR data record patient features, treatment interventions, and achieved treatment outcomes. For each patient sample *u*, we observe a set of patient features ***x***_u_, a set of treatment interventions ***a***_u_ conditioned on ***x***_u_, and the achieved treatment outcome *y*_*u*_. The EHR dataset can be described as,

1 $$ \mathcal{D}=\left\{\left({\boldsymbol{x}}_{\mathrm{u}},{\boldsymbol{a}}_{\mathrm{u}},{y}_u\right)|\mathrm{u}=1,\cdots, {\mathrm{N}}_{\mathcal{D}}\right\} $$

We propose the ADTEP model to address the aforementioned problem. The ADTEP inherits the loss function of traditional classification models, and takes advantage of the adversarial learning scheme to extract representative and discriminative features, which not only semantically encode the essential and critical information contained in the patient EHR, but also provide the benefit of achieving high accuracy for TEP.

**Fig. 1:**
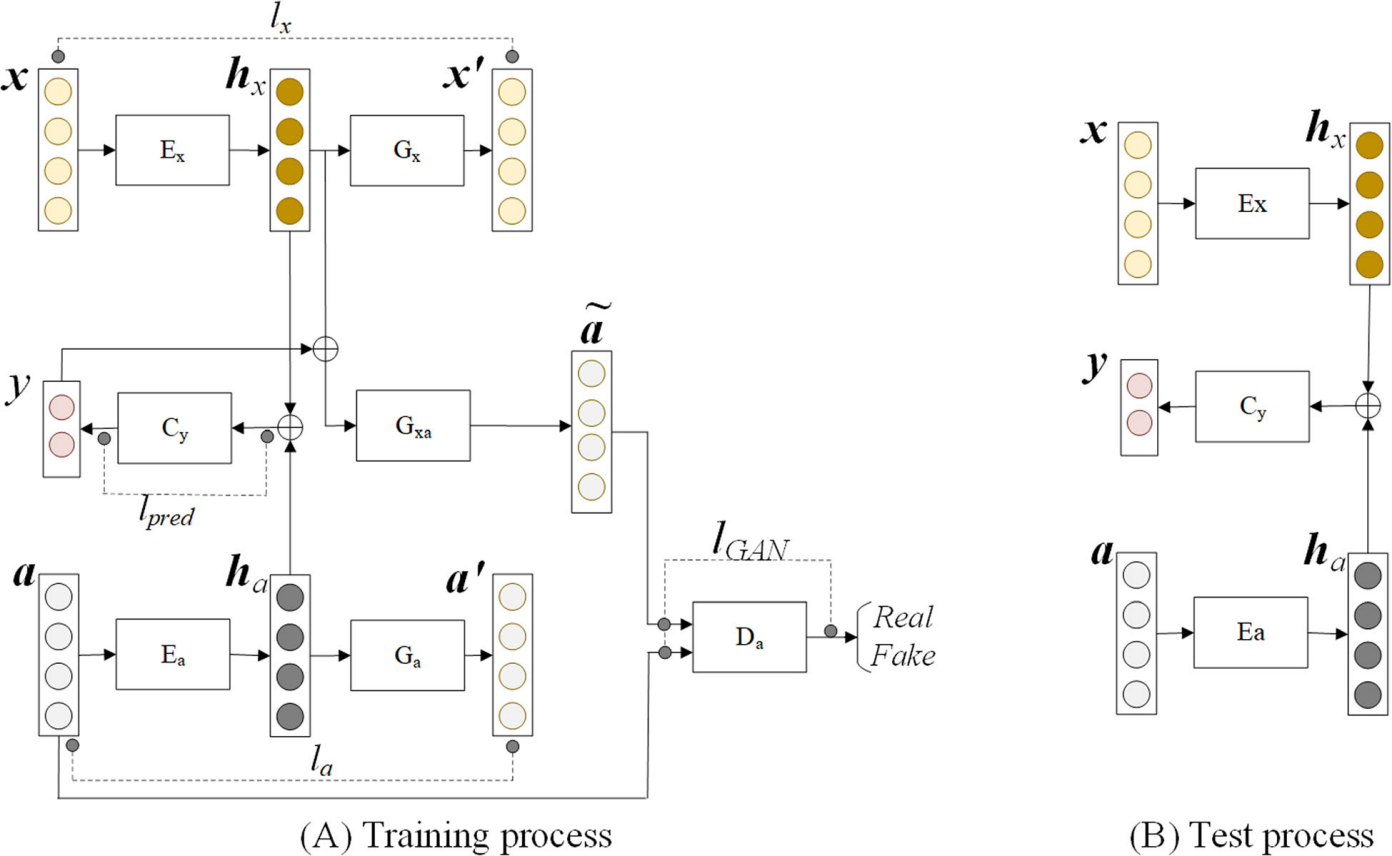
Adversarial deep treatment effect prediction (ADTEP) model

As illustrated in Fig. 1(A), during the training process, the proposed ADTEP contains seven components: a patient feature encoder E_x_, a treatment intervention encoder E_a_, a patient feature decoder G_x_, a treatment intervention decoder G_a_, a treatment intervention generator G_xa_, a treatment intervention discriminator D_a_, and a logistic regression layer for TEP C_y_. In detail, given a patient sample (***x***, ***a***, *y*), two encoder layers E_x_ and E_a_ are first employed to extract the latent features ***h***_*x*_ and ***h***_*a*_ from ***x*** and ***a***, respectively. The reconstructed features ***x***^′^ and ***a***^′^ can then be estimated from the latent features ***h***_*x*_ and ***h***_*a*_, using the decoders G_x_ and G_a_. Note that E_x_ and G_x_ form an AE for patient feature observations, and for E_a_ and G_a_ to reconstruct treatment interventions. Both AEs E_x_- G_x_ / E_a_- G_a_ are adopted to capture robust and discriminative patient feature/treatment representations in the latent feature vector ***h***_x_ / ***h***_a_. Consequently, the latent feature vectors ***h***_x_ and ***h***_a_ are concatenated to form the input of C_y_ for TEP.

As treatment interventions are performed conditioned on patient features in clinical practice, we feed the latent patient features ***h***_*x*_ into another generator G_xa_ to yield treatment interventions $\overset{\sim }{\boldsymbol{a}}$**,** conditioned on the treatment outcome *y* of the patient sample $\overset{\sim }{\boldsymbol{a}}={\mathrm{G}}_{xa}\left({\boldsymbol{h}}_x,y\right)$, and then use a discriminator to distinguish whether or not the generated treatment interventions $\overset{\sim }{\boldsymbol{a}}$ and original ones ***a*** originate from the same treatment distributions. The use of the generator G_xa_ allows us to learn the latent correlations between patient features and treatments. This learning strategy can regularize the latent features ***h***_*x*_ to encode most of the information shared between the patient characteristics and subsequent treatments. The details are as follows.

### Encoder-decoder

We employ two AEs, namely E_x_- G_x_, and E_a_- G_a_, to learn the latent representations of patient characteristics and treatments, respectively. A simple form of an AE is a feed-forward and non-recurrent neural network [24, 38], consisting of an input layer, an output layer and one or multiple hidden layers in between. The AE attempts to reconstruct the input from the corrupted data. Formally, given an M-dimensional input patient feature vector ***x*** ∈ *ℝ*^M^, it is mapped to the code vector ***h***_*x*_ with the encoding function E_*x*_(W_e_***x*** + b_e_), and subsequently during the decoding step, it maps the code vector ***h***_*x*_ to the output vector ***x***^′^, which reconstructs the input vector with the decoding function G_*x*_(*W*_*d*_***h***_*x*_ + *b*_*d*_), where W_e_ ∈ *ℝ*^K × M^ and W_d_ ∈ *ℝ*^M × K^ are weighted matrices, *b*_e_ ∈ *ℝ*^K^ and *b*_*d*_ ∈ *ℝ*^M^ are the corresponding bias terms, E_*x*_(·) and G_*x*_(·) are nonlinear activation functions, and K is the number of nodes in the hidden layer.

Similar to the AE E_x_- G_x_, the treatment encoder E_a_ takes the treatment vector ***a*** as input and generates the latent treatment vector ***h***_*a*_, which is subsequently fed into decoder G_a_ to generate the reconstructed treatment ***a***^′^. Both E_a_ and G_a_ constitute a treatment AE, which aims at reconstructing the treatment behavior from the patient EHR data.

It is very challenging to generate the treatment vector $\overset{\sim }{\boldsymbol{a}}$ of a patient sample from his or her clinical status representation $\boldsymbol{x}:P\left(\overset{\sim }{\boldsymbol{a}}|\boldsymbol{x}\right)$, owing to the large appearance variations in the treatment selections given the patient characteristics in clinical settings. To address this problem, we use the patient feature encoder E_*x*_ and treatment generator G_*xa*_ to form an AE. Specifically, given a patient feature vector ***x*** and the known treatment effect *y*, E_*x*_ is adopted to extract the latent feature vector ***h***_x_ ***=*** E_*x*_(***x***). The feature vector ***h***_x_ is expected to encode the correlational information between the patient characteristics and treatments after adversarial training, and the treatment vector $\overset{\sim }{\boldsymbol{a}}$ can be estimated from the latent feature vector ***h***_x_, using G_*xa*_ conditioned on the obtained treatment effect $\mathrm{y}:\overset{\sim }{\boldsymbol{a}}={\mathrm{G}}_{xa}\left({\boldsymbol{h}}_x,y\right)$.

### Patient feature reconstruction loss

In this study, we measure the reconstruction performance for patient feature ***x*** conducted by the encoder E_x_ and decoder G_x_. For efficient learning of the encoder-decoder, standard practice is to use the Euclidean distance between the input and the generated output to minimize the patient feature reconstruction loss, that is,

2 $$ {\mathcal{L}}_x={\mathbbm{E}}_{\boldsymbol{x},\boldsymbol{a},y\sim {P}_{data}\left(\boldsymbol{x},\boldsymbol{a},y\right)}{\left|\left|\boldsymbol{x}-{\mathrm{G}}_x\left({\mathrm{E}}_x\left(\boldsymbol{x}\right)\right)\right|\right|}_2^2 $$

Here, the encoder E_x_ maps the input patient feature vector ***x*** into the latent one ***h***_*x*_, and then, the decoder G_x_ reconstructs the feature ***x***^′^ from ***h***_*x*_.

### Treatment reconstruction loss

The reconstruction performance for treatment vector ***a*** is measured by means of the encoder E_a_ and decoder G_a_. Similarly to the patient feature reconstruction loss ${\mathcal{L}}_x$, the treatment reconstruction loss ${\mathcal{L}}_a$ can be measured as follows:

3 $$ {\mathcal{L}}_a={\mathbbm{E}}_{\boldsymbol{x},\boldsymbol{a},y\sim {P}_{data}\left(\boldsymbol{x},\boldsymbol{a},y\right)}{\left|\left|\boldsymbol{a}-{\mathrm{G}}_a\left({\mathrm{E}}_a\left(\boldsymbol{a}\right)\right)\right|\right|}_2^2 $$

Minimizing Eqs. (2) and (3) aids us in determining a representative latent feature space for the patient clinical characteristics and subsequent treatments.

### Discriminator

As a popular learning formulation for deep learning, adversarial learning is similar to a competition game, in which a discriminator judges a data sample as real or fake; in contrast, a generator attempts to produce indistinguishable samples without being detected [17, 25, 39]. Inspired by adversarial learning and based on the common sense whereby treatments are conditioned on patient characteristics in a clinical context [10], we encourage the reconstruction of treatments from discriminative patient features that are similar to real ones, so that the prediction performance can be enriched.

To this end, we design a treatment discriminator D_a_ to differentiate the reconstructed treatment vector $\overset{\sim }{\boldsymbol{a}}$ from the true observed treatment ***a***. In particular, we employ a binary classifier to categorize the given input as “real” if the input is the actual treatment vector performed on patients, and “fake” otherwise. D_a_ enables the proposed model to learn a hidden treatment representation ***h***_*a*_ from the EHR data. Meanwhile, D_a_ causes the latent patient features ***h***_*x*_ to be treatment specific. As a result, it improves the discriminative capability of the learned features, and makes them particularly optimized for TEP.

### Adversarial loss

${\mathcal{L}}_{GAN}$ is optimized to train the encoder E_x_, decoder G_*xa*_, and discriminator *D*_*a*_. The encoder E_x_ is trained to generate the treatment-specific patient feature ***h***_*x*_, while the decoder G_*xa*_ is trained to generate treatments conditioned on ***h***_*x*_ manipulated by the treatment outcome label *y*. The discriminator D_*a*_ attempts to distinguish the actual treatment vector ***a*** as real and the reconstructed one $\overset{\sim }{\boldsymbol{a}}$ as fake. We define the adversarial loss ${\mathcal{L}}_{GAN}$ as:

4 $$ {\mathcal{L}}_{GAN}={\mathbbm{E}}_{\boldsymbol{x},\boldsymbol{a},y\sim {p}_{data}\left(\boldsymbol{x},\boldsymbol{a},y\right)}\left[\log {\mathrm{D}}_a\left(\boldsymbol{a}\right)\right]+{\mathbbm{E}}_{\boldsymbol{x},\boldsymbol{a},y\sim {p}_{data}\left(\boldsymbol{x},\boldsymbol{a},y\right)}\left[\log \left(1-{\mathrm{D}}_a\left({\mathrm{G}}_{xa}\left({\mathrm{E}}_x\left(\boldsymbol{x}\right),y\right)\right)\right)\right]. $$

### Treatment outcome predictor

Given a testing patient sample with patient feature vector ***x***, treatment vector ***a*** conditioned on ***x***, and an unknown treatment outcome label y, we can learn the representative and informative features ***h***_x_ and ***h***_a_ with respect to the patient characteristics, and subsequently the treatments performed on the patient, respectively, and then concatenate these as [***h***_x_, ***h***_a_] to be fed into the treatment effect predictor C_y_, so that treatment effects can be estimated for the target patient.

### Treatment outcome prediction loss

${\mathcal{L}}_{pred}$. In this study, we employ a logistic regression layer for the treatment outcome prediction, in which the input is the concatenation of the latent patient feature vector ***h***_*x*_ and treatment vector ***h***_a_. This is used to estimate the treatment effect of patient samples given their clinical conditions and performed treatment interventions. The loss can be measured using cross-entropy as follows:

5 $$ {\mathcal{L}}_{pred}=\frac{1}{N_D}{\sum}_{u=1}^{N_D}L\left({W}_{pred},{b}_{pred};{\boldsymbol{x}}_u,{\boldsymbol{a}}_u,{y}_u\right)=\frac{1}{N_D}{\sum}_{u=1}^{N_D}\left({y}_u\log {y}_u^{\prime }+\left(1-{y}_u\right)\log \left(1-{y}_u^{\prime}\right)\right), $$

where *y*^′^ is the predicted treatment outcome.

### Model learning

As demonstrated in the section above, our training is defined by four loss functions: 1) loss of GAN ${\mathcal{L}}_{GAN}$, loss of patient feature reconstruction ${\mathcal{L}}_x$, loss of treatment reconstruction ${\mathcal{L}}_a$, and loss of treatment outcome prediction ${\mathcal{L}}_{pred}$. In summary, the objective function of the ADTEP is expressed as:

6 $$ \underset{E_x,{E}_a,{G}_x,{G}_a,{G}_{xa},{C}_y}{\min}\underset{D_a}{\max }{L}_{pred}+\alpha \left({L}_x+{L}_a\right)+\beta {L}_{GAN}, $$

where *α* and *β* are trade-off parameters for balancing the importance of the corresponding components.

The learning algorithm of the proposed model can be formulated as follows:

1. Update the parameters of the patient feature encoder and decoder $\left\{{\Theta}_{E_x},{\Theta}_{G_x}\right\}$ by minimizing the patient feature reconstruction loss ${\mathcal{L}}_x$. Note that the encoder *E*_*x*_ and decoder *G*_*x*_ are trained to reconstruct patient characteristics. Moreover, the encoder *E*_*x*_ is regularized to generate treatment-specific patient characteristics, as it also needs to generate treatments, as discussed previously.
2. Update the parameters of the treatment encoder and decoder $\left\{{\Theta}_{E_a},{\Theta}_{G_a}\right\}$ by minimizing the treatment reconstruction loss ${\mathcal{L}}_a$.
3. Update the discriminator parameter {Θ_*d*_} to optimize ${\mathcal{L}}_{GAN}$ by maximizing the adversarial loss $\underset{D}{\max }{\mathcal{L}}_{GAN}$.
4. Update the treatment effect predictor {Θ_*c*_} by minimizing the prediction loss $\underset{C}{\min }{\mathcal{L}}_{pred}$.

Note that the above objectives are optimized in an iterative manner. Specifically, E_*x*_, E_*a*_, G_*x*_, G_*a*_, G_*xa*_, D_a_, and C_*y*_ improve one another during the alternative training process. With D_*a*_ being more capable of distinguishing the generated fake treatment vector and real one, G_*xa*_ encourages the generation of fake treatments which based on patient feature to compete with the discriminator D_*a*_. To this end, the encoder E_*x*_ and decoder G_x_ are driven to encode the representative patient features into the latent feature vector ***h***_*x*_. Thereafter, the treatment generator G_*xa*_ learns how to map the latent patient feature ***h***_*x*_ to conditioned treatments $\overset{\sim }{\boldsymbol{a}}$ corresponding to the input patient feature ***x***. This process makes the features particularly optimized for TEP.

Fig. 1 (B) presents the flowchart of the TEP test process. In particular, the AEs E_*x*_- G_*x*_ and E_a_- G_*a*_ are used to generate the latent feature representations ***h***_*x*_ and ***h***_*a*_, which are then concatenated as the input of C_*y*_ to predict treatment outcomes for the test patient samples

### Treatment effect analysis for target outcome

To analyze the association between the treatment and clinical outcome in an interpretable manner, we compute the effect of each treatment for the target outcome following training. We firstly compute the mean loss ${\mathcal{L}}_{pred}$ over the training samples. Thereafter, for each treatment *k*, 1 ≤ *k* ≤ K, and for each patient sample *u*, 1 ≤ *u* ≤ *N*_*D*_, we let ${\hat{\boldsymbol{a}}}^{(u)}={\boldsymbol{a}}^{(u)}$ and then set ${\hat{\boldsymbol{a}}}_k^{(u)}=0$. Based on the adjusted ${\hat{\boldsymbol{a}}}^{(u)}$, we compute the mean loss, as follows:

7 $$ {\mathcal{L}}_{pred}^k:{\mathcal{L}}_{pred}=\frac{1}{N_D}{\sum}_{u=1}^{N_D}L\left({W}_{pred},{b}_{pred};{\boldsymbol{x}}_u,{\hat{\boldsymbol{a}}}^{(u)},{y}_u\right), $$

and then compute the effect of treatment *k* for the target outcome:

8 $$ \mathrm{ef}{\mathrm{f}}^k={\mathcal{L}}_{pred}^k-{\mathcal{L}}_{pred} $$

Note that the calculated value of eff^*k*^ discloses the relevant treatment for the target variation, which is helpful for physicians to understand whether the performed treatment has an effect on the target outcome, and the means by which the black-box deep learning-based TEP model operates in a reasonable and trustworthy manner. We argue that the analysis results can provide certain insights for the formation of treatment effects on the target clinical outcomes.

## Experiments

We conducted two clinical case studies in cooperation with the Cardiology Department of the Chinese PLA General Hospital. The first investigated major adverse cardiac event (MACE) prediction after acute coronary syndrome (ACS), while the second focused on one-year readmission prediction for heart failure (HF) patients, as detailed in the following subsections.

Note that categorical features, such as gender, operation, medicine and complication, are represented as binary values. Meanwhile, continuous features, such as age, BMI and lab test values, are categorized into three levels: lower than normal, normal and higher than normal, according to the clinical protocol adopted by the hospital, and represented as one-hot vectors with three dimensions.

All experiments were conducted on a Microsoft Surface Pro 5 Compatible PC with an Intel Core i7-7660U CPU 2.50 GHz and 8 GB of main memory, running on Microsoft Windows 10. The proposed model was implemented in Python, and the source code is available at https://github.com/ZJU-BMI/treatment. Prior approval for conducting the study was obtained from the data protection committee of the hospital. We wish to make it clear that the patient data were anonymized in this study and in this paper.

### Performance comparisons

To demonstrate the effectiveness of our proposed model, we compare the proposed ADTEP with: the proposed model without adversarial learning, namely the DTEP model. For the DTEP, we use AEs to generate the latent representations of both the patient characteristics and the subsequent treatments, concatenate the derived latent features, and then feed the obtained feature vector into a logistic regression layer, yielding a TEP model. Note that DTEP does not consider the correlations between the patient state and the treatment. Moreover, we compare the proposed model to benchmark models using the experimental datasets, including logistic regression (LR) and the support vector machine (SVM). L2-regularization is used in LR, DTEP and ADTEP. We search the best values of hyper-parameters with grid search strategy and all the results shown in this paper are obtained on the condition of the best settings.

### Evaluation metrics

The performance was evaluated by the Area Under the receiver operating characteristic (ROC) curve (AUC), accuracy, precision, recall and F1 score. To estimate the performance of the treatment effect estimation in a less biased manner than single-round testing, we repeated the experiments five times to validate the performance of each model on the experimental dataset. Furthermore, the five-fold cross-validation strategy was applied in each run of the experiment. As a result, we obtained a group of experimental results for each model, on which the mean value and confidence intervals were calculated.

### ACS case study

#### Data description

ACS refers to a group of conditions resulting from decreased blood flow in the coronary arteries, whereby that part of the heart muscle is unable to function properly or dies [40]. The basic treatment principles are the same for all types of ACS; however, several important aspects of treatment depend on the specific characteristics of ACS patients. For example, the comorbidities of ACS patients, presence or absence of elevation of the ST segment on the electrocardiogram, and different treatment interventions may result in varying treatment effects [41–43]. To this end, the ability to leverage a quantitative paradigm for alleviating adverse treatment effects and improving patient outcomes, in terms of both prediction and prevention could potentially deliver significant benefits to both patients and their families, as well as society. Regarding the indicators of treatment effects for ACS patient samples, we select the MACE after ACS as the label for treatment effects. MACE is a typical indicator of the treatment effect, and it often occurs suddenly, resulting in high mortality and morbidity [12, 44]. In clinical practice, MACE has a significant impact on clinical decision-making for ACS patient care and treatment.

To conduct the ACS case study, we collaborated with the clinicians of the cardiology department, and extracted a collection of 3463 ACS patient samples from the hospital EHR system. The dataset documented 326 patient features including demographics, operations, medications, laboratory values and diagnosis, etc. Specifically, for features with multiple measurement, like laboratory values, we kept the initial measurement on admission. Preprocessing was conducted on the collected ACS dataset. In particular, both patient samples and variables with more than 30% of missing values were excluded from the analysis. Other than this, no further efforts were made to handle the missing data in the experiments. As a result, 2930 patient samples with a median age of 62.27 years were obtained, among which 2080 (71%) were female. A summary of the statistics of the dataset is provided in Table 1, where shows the information of several important patient characteristics selected by our clinical collaborates. Note that the *P*-values of features with continues values were calculated by Mann-Whitney U test, while the P-values of features with binary values were calculated by Chi-squared test.

**Table 1:** Baseline characteristics of experimental ACS dataset

Characteristics	No. of participants (*n* = 2930)	MACE (*n* = 752)	Non-MACE (*n* = 2178)	P-value
Age (years), mean (SD)	62.27 ± 12.11	67.12 ± 11.95	60.60 ± 11.71	< 0.001
Female sex (T/F)	2080/850	528/225	1552/625	0.573
Hypertension (T/F)	1981/949	537/215	1444/734	0.011
Diabetes mellitus (T/F)	1986/803	482/224	1504/439	< 0.001
Hypercholesterolemia (T/F)	2362/568	623/129	1739/439	0.082
Previous PCI (T/F)	816/2114	214/538	602/1576	0.701
Previous CABG (T/F)	86/2844	38/714	48/2130	< 0.001
ST-segment elevations ECG (T/F)	106/2824	27/725	79/2099	0.947
BMI (kg/m^2^), mean (SD)	25.90 ± 11.30	25.50 ± 12.73	26.03 ± 10.76	0.333
CCR (ml/min/ m^2^), mean (SD)	78.73 ± 38.19	85.36 ± 48.30	76.41 ± 33.65	< 0.001
CKMB (umol/L), mean (SD)	9.49 ± 14.95	9.30 ± 11.45	9.56 ± 16.09	0.715
Treatment				
Coronary angiography (T/F)	993/1937	270/482	723/1455	0.191
Nitroglycerin (T/F)	904/2026	292/460	612/1566	< 0.001
Vasodilator (T/F)	951/1979	305/447	646/1532	< 0.001
Antihypertensive therapy (T/F)	1375/1555	385/367	990/1188	< 0.001
Hypoglycemic therapy (T/F)	451/2479	127/625	324/1854	0.208
Lipid lowering therapy (T/F)	511/2419	129/623	382/1796	0.854
Blood transfusion (T/F)	91/2839	28/724	63/2115	0.312
Quick-acting rescue (T/F)	796/2134	236/516	560/1618	< 0.001
Aspirin (T/F)	730/2200	204/548	526/1652	0.114
Antiarrhythmia (T/F)	114/2816	45/707	69/2109	< 0.001
Anti-angina (T/F)	1216/1714	376/376	840/1338	< 0.001
Antiplatelet (T/F)	933/1997	255/497	678/1500	0.172

BMI: body mass index; CABG: coronary artery bypass grafting; CCR: Creatinine clearance; CKMB: creatine kinase MB; ECG: electrocardiogram; PCI: percutaneous coronary intervention; SD: standard deviation

#### Experimental results and analysis

**Fig. 2:**
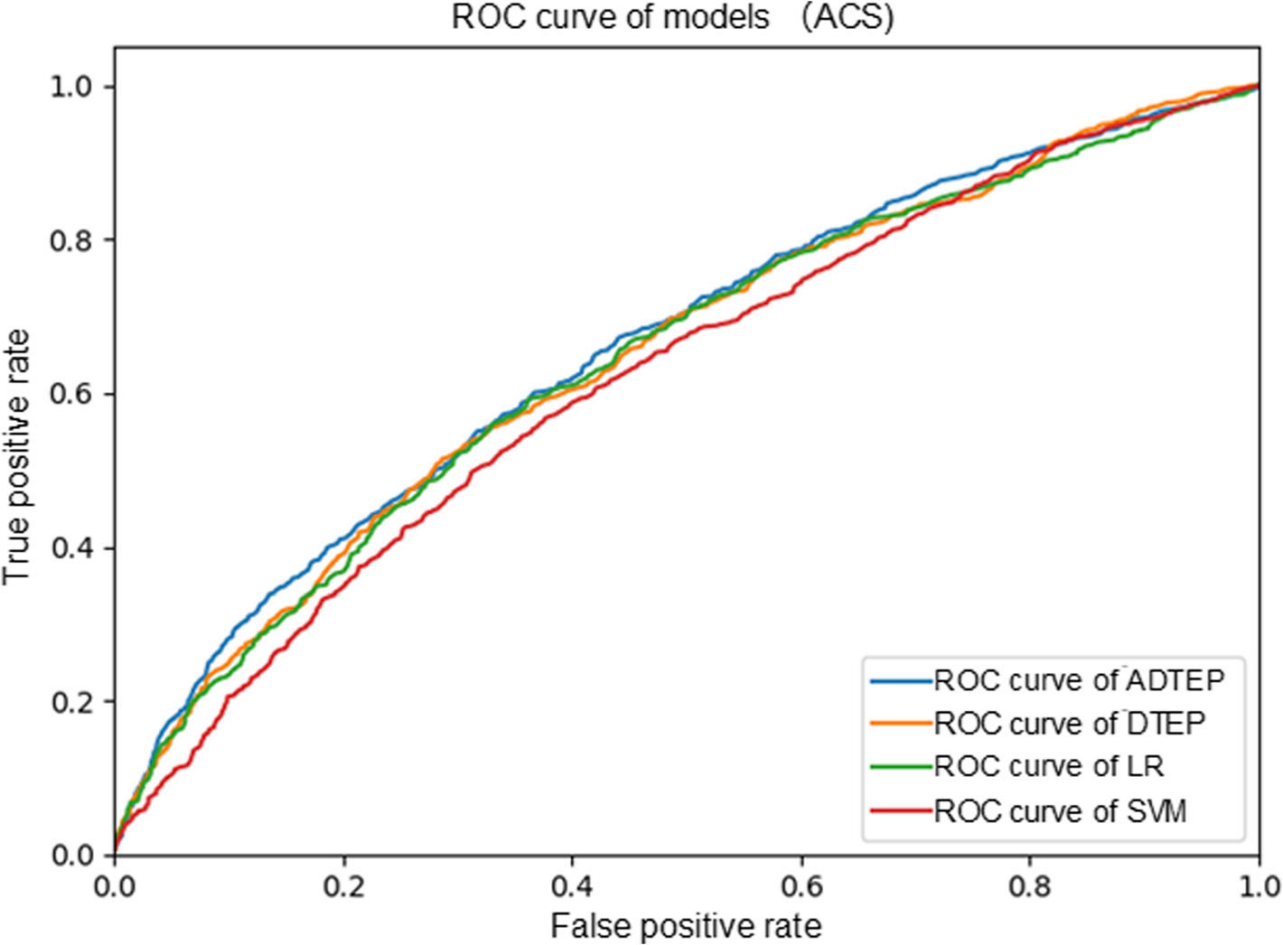
ROC curves for MACE prediction after ACS

Table 2 presents the TEP performance achieved on the experimental ACS dataset. As can be observed from Table 2, the proposed model achieved superior performance compared to benchmark models on the experimental dataset. ADTEP performed slightly better than DTEP in terms of both the AUC and F1. Although DTEP outperformed ADTEP in terms of the average accuracy, the performance gain was marginal. These findings indicate that the incorporation of correlational information between patient characteristics and treatments by means of the adversarial learning strategy was useful in predicting the treatment effects of ACS patient samples. Figure 2 illustrates the ROC curves for MACE prediction after ACS, also demonstrating that the proposed ADTEP achieved comparative performance with benchmark models. In particular, ADTEP exhibited 1.4, 2.2, and 6.3% performance gains for MACE prediction in terms of AUC over DTEP, LR, and SVM, respectively.

**Table 2:** Experimental results for accuracy, AUC, precision, recall and F1 score on ACS experimental dataset

Method	Accuracy (mean ± SD)	AUC (mean ± SD)	Precision (mean ± SD)	Recall (mean ± SD)	F1 score (mean ± SD)
LR	0.744 ± 0.016	0.648 ± 0.026	0.505 ± 0.078	0.198 ± 0.034	0.284 ± 0.044
SVM	0.716 ± 0.010	0.621 ± 0.014	0.402 ± 0.032	**0.219** ± 0.026	0.283 ± 0.027
DTEP	**0.747** ± 0.010	0.653 ± 0.021	**0.524** ± 0.056	0.181 ± 0.025	0.268 ± 0.031
ADTEP	0.746 ± 0.012	**0.662** ± 0.020	0.515 ± 0.058	0.210 ± 0.036	**0.297** ± 0.042

**Fig. 3:**
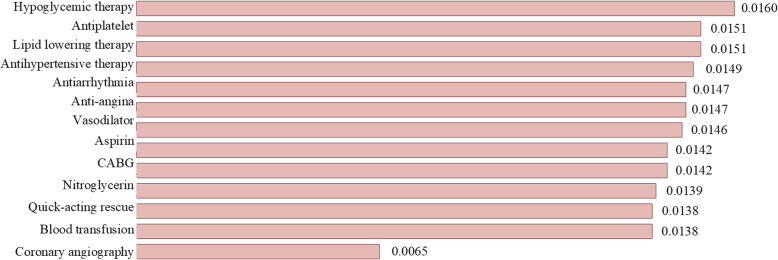
Achieved *Eff*^*k*^ values of treatments on ACS dataset

Figure 3 displays the measured *Eff*^*k*^ values of treatments on the ACS dataset. Two of the three most relevant treatments for MACE prediction were found to be: antiplatelet and lipid lowering therapy, which are consistent with existing medical knowledge as major interventions for ACS [45, 46]. The most irrelevant treatment for MACE was found to be coronary angiography. This finding is also reasonable, because coronary angiography is not a specific treatment for relieving the symptoms of ACS, but rather a procedure to determine how blood flows through the arteries in the hearts, and thus, is less relevant to influencing the occurrence of MACE after ACS. Surprisingly, we found that hypoglycemic therapy had the strongest correlation with MACE, while nitroglycerin had a less significant correlation. This is inconsistent with clinical guidelines as hypoglycemic therapy is mainly adopted for the treatment of type II diabetes, while nitroglycerin is recognized as a major treatment for preventing ischemic events after ACS. These findings may contain suggestive hypotheses that could be validated by further clinical investigations.

### HF case study

#### Experimental setup

HF is a complex clinical syndrome that affects at least 40 million people globally and is increasing in prevalence [47]. Although not all conditions leading to HF can be reversed, treatments can improve the signs and symptoms of HF and help patients to live longer. Usually, several HF-specific treatments are available, such as angiotensin converting enzyme inhibitor (ACEI)/angiotensin receptor blocker (ARB), beta-blockers and aldosterone antagonists, and it is meaningful to select appropriate treatments for an individual HF patient according to his or her clinical conditions and the desired treatment effects. The objective of this case study was to analyze the effects of treatments on the one-year readmission of HF patients.

The experimental dataset consisted of 736 HF patients with one-year follow up information (461 readmitted, 275 not readmitted). Each patient sample contained 105 features including demographics (such as age, gender), vital signs (including blood pressure and heart rate), laboratory tests (for example, creatinine kinase (CK), cardiac troponin T (cTnT)), echocardiography (such as ejection fraction), comorbidities (for example, diabetes and renal insufficiency), and treatments (including ACEI, ARB, and beta-blockers) adopted for these patients. Specifically, for features with multiple measurement, like vital signs and laboratory values, we kept the initial measurement on admission. Table 3 lists the information of several important patient characteristics suggested by our clinical collaborators based on their knowledge about HF. As the same with Table 1, the *P*-values of features with continues values were calculated by Mann-Whitney U test, while the P-values of features with binary values were calculated by Chi-squared test.

**Table 3:** Baseline characteristics of experimental HF dataset

Characteristics	No. of participants (*n* = 736)	Readmission in one year (*n* = 461)	Non-readmission in one year (*n* = 275)	P-value
Age (years), mean (SD)	64.29 ± 13.55	63.66 ± 13.55	65.34 ± 13.53	0.104
Female sex (T/F)	508/227	331/130	177/97	0.050
Hypertension (T/F)	526/210	323/138	203/72	0.314
Diabetes mellitus (T/F)	466/270	283/178	183/92	0.185
Renal insufficiency (T/F)	592/144	359/102	233/42	0.030
SBP (mmHg), mean (SD)	133.41 ± 20.41	130.33 ± 20.02	138.57 ± 20.04	< 0.001
DBP (mmHg), mean (SD)	77.13 ± 13.66	76.15 ± 13.86	78.76 ± 13.19	0.012
Heart rate (b.p.m) mean (SD)	79.98 ± 16.37	81.17 ± 17.01	78.00 ± 15.06	0.011
Creatinine (umol/L), mean (SD)	100.35 ± 64.5	106.77 ± 72.85	89.61 ± 45.50	< 0.001
LVEF (%), mean (SD)	43.74 ± 11.86	41.92 ± 12.12	46.80 ± 10.76	< 0.001
CK (umol/L), mean (SD)	87.79 ± 82.04	89.71 ± 80.56	84.60 ± 84.50	0.414
cTnT (ng/ml), mean (SD)	0.058 ± 0.38	0.077 ± 0.47	0.025 ± 0.057	0.068
Treatment				
Diuretics (T/F)	536/200	344/117	202/73	< 0.001
ACEI (T/F)	442/294	279/182	163/112	0.797
ARB (T/F)	480/256	296/165	184/91	0.507
Beta-blocker (T/F)	588/148	367/94	221/54	0.879
CCB (T/F)	454/282	307/154	147/128	< 0.001
Statin (T/F)	536/200	322/139	214/61	0.023
Digoxin (T/F)	457/279	257/204	200/75	< 0.001
Nitrates (T/F)	454/282	274/187	180/95	0.122
Aspirin (T/F)	513/223	314/147	199/76	0.258
Clopidogrel (T/F)	379/357	244/217	140/135	0.650
Warfarin (T/F)	638/98	399/62	239/36	0.979
Spironolactone (T/F)	402/334	288/173	161/114	0.328
Antibiotics (T/F)	713/23	446/15	267/8	0.967
Antiacid (T/F)	589/147	367/94	222/53	0.786

ACEI: angiotensin-converting enzyme inhibitor; ARB: angiotensin receptor blocker; CCB: calcium channel blocker; cTnT: cardiac troponin T; CK: creatinine kinase; DBP: diastolic blood pressure; LVEF: left ventricular ejection fraction; SBP: systolic blood pressure

#### Experimental results and analysis

Table 4 reports the experimental results on the HF dataset. It can be observed that the proposed ADTEP outperformed benchmark models in terms of both accuracy and AUC. Specifically, ADTEP exhibited boosted performance compared to the benchmark models. This finding indicates that the proposed model can extract more discriminative representations from EHR data for predicting the treatment effects of HF patients, by using deep learning tactics. Moreover, by introducing the adversarial learning strategy, the proposed ADTEP obtained performance gains of 4.8 and 4.1% in terms of accuracy and AUC, respectively, compared to DTEP. This demonstrates that discriminative representations can be obtained for efficient treatment effect estimation by extracting correlational information between patient characteristics and subsequent treatments.

**Table 4:** Experimental results for accuracy, AUC, precision, recall and F1 score on experimental HF dataset

Method	Accuracy (mean ± SD)	AUC (mean ± SD)	Precision (mean ± SD)	Recall (mean ± SD)	F1 score (mean ± SD)
LR	0.647 ± 0.030	0.682 ± 0.039	0.677 ± 0.022	0.836 ± 0.037	0.748 ± 0.021
SVM	0.642 ± 0.034	0.633 ± 0.027	0.669 ± 0.018	**0.849** ± 0.050	0.748 ± 0.028
DTEP	0.624 ± 0.034	0.661 ± 0.038	0.679 ± 0.055	0.830 ± 0.149	0.721 ± 0.064
ADTEP	**0.654** ± 0.025	**0.688** ± 0.040	**0.680** ± 0.019	0.848 ± 0.034	**0.754** ± 0.019

**Fig. 4:**
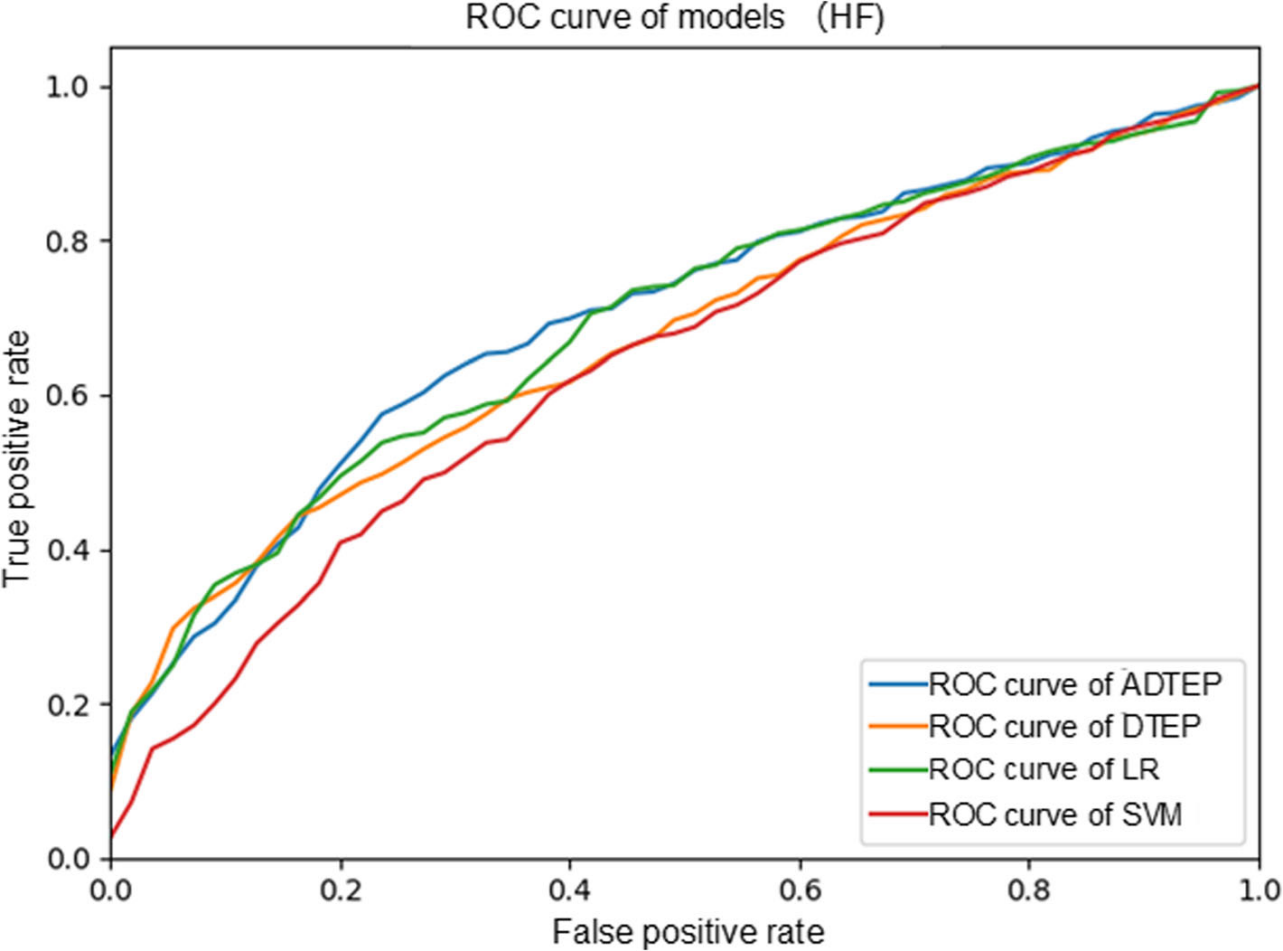
ROC curves for one-year readmission prediction for HF patients

Figure 4 illustrates the ROC curves achieved by both the proposed model and baseline approaches on the HF dataset. As can be observed from Fig. 4, the proposed ADTEP performed better than benchmark models. In particular, the proposed ADTEP exhibited performance gains of over 4.1, 0.9, and 8.7% in terms of the AUC in comparison with DTEP, LR, and SVM, respectively, on the experimental dataset, although LR curve closely approached the ADTEP curve. These observations indicate that deep learning tactics can indeed extract representative and discriminative features from data and therefore aid in achieving comparable TEP performance compared to state-of-the-art models. When comparing ADTEP and DTEP, it was observed that ADTEP outperformed DTEP in terms of the ROC curve. This indicates that incorporating adversarial learning into the TEP can extract more representative features to improve the TEP performance.

**Fig. 5:**
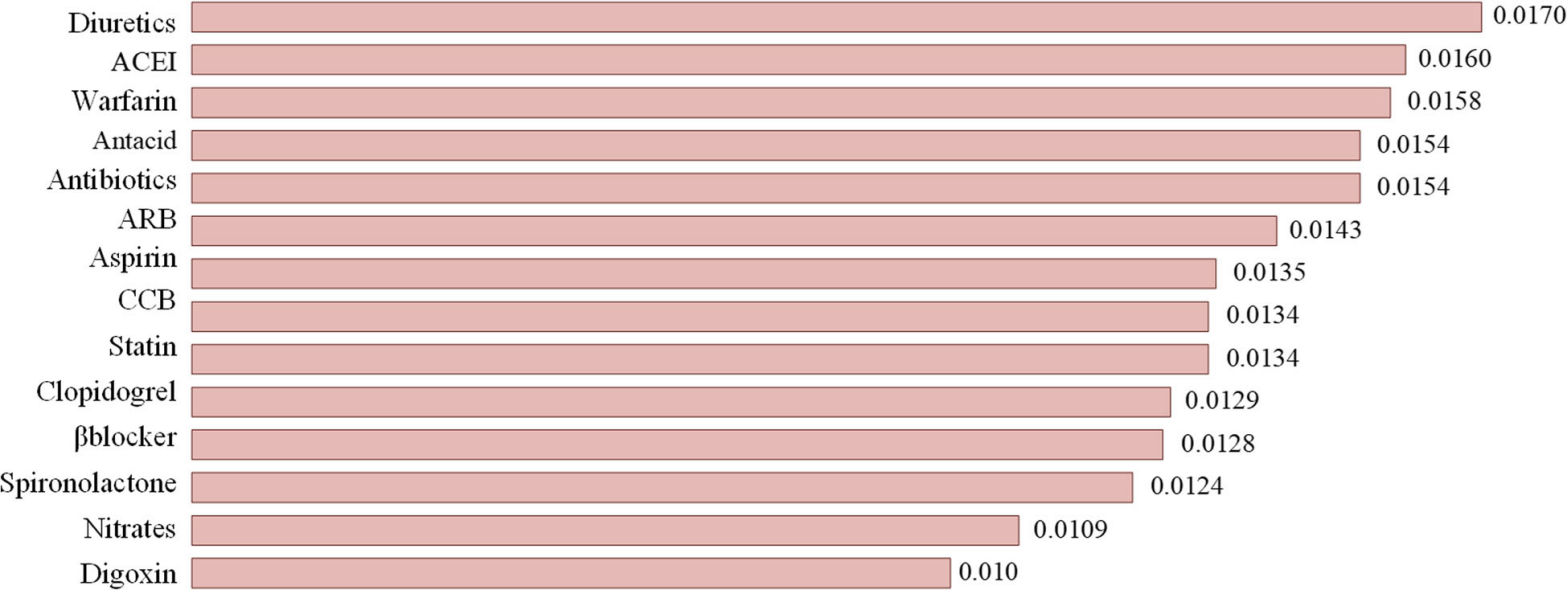
Achieved *Eff*^*k*^ values of treatments on HF dataset

Moreover, to analyze the correlations between the treatments and clinical outcomes, we used Eq. (8) to measure the *Eff*^*k*^ values of the treatments on the target outcome (that is, one-year readmission), based on the HF dataset. As can be observed from Fig. 5, the most relevant treatments for the target outcome were: diuretics, AECI, and Warfarin, which is consistent with existing medical domain knowledge, as these are the main adopted medications for HF [47]. In contrast, the least relevant treatment for the target outcome of HF was Digoxin. Note that this finding is also consistent with the newly published clinical guidelines because Digoxin is a type of obsolete medications for HF therapy and may increase the risk of bleeding of HF patients [48]. This finding may contain suggestive hypotheses that could be validated by further clinical investigations.

## Discussion

Overall, compared to benchmark approaches, our model can improve the TEP performance in terms of two aspects. Firstly, we use deep learning models to generate latent representations of patient features and treatments. This can extract deep information from heterogeneous EHR data. Secondly, the expression of adversarial learning extracts abundant latent and nonlinear correlations between patient status and corresponding treatments, so that precisely representative features can be extracted from the data. With such ability, our proposed model shows superiority against other models on experimental results. Moreover, the results validate our assumption that the correlational information between patient characteristics and treatments can indeed improve the TEP performance. Furthermore, our model can extract informative treatments given the target outcome. Several of these extracted treatments are not only consistent with existing medical knowledge, but also contain suggestive hypotheses that could be validated by further investigations in the medical domain.

The experimental results were evaluated by hospital managers and clinical experts at the Chinese PLA General Hospital, who understand the beneficial effects of the proposed model. They indicated the potential of applying the proposed model in clinical practice for efficient treatment selection and improvement. Specifically, the proposed model can be utilized to support clinical decision-making and aid in treatment adoption. For example, the patient characteristics can be analyzed to aid healthcare professionals in scheduling individual treatment interventions for patients, in order to achieve the expected treatment effects. The method is also applicable to clinical decision support systems that recommend appropriate treatment interventions matching the specific patient statuses. This could guide healthcare professionals to schedule appropriate treatment interventions based on the measurement of the target patient statuses and the desired treatment effects, by meaningfully employing a large volume of EHR data to derive non-trivial knowledge explaining the treatment intentions and behaviors. In this regard, our clinical collaborators advocate us to develop and deploy a TEP service in the EHR system. Such a service will not only predict treatment effects nearly at run-time in the treatment processes of patients, but also essentially assist healthcare professionals to schedule appropriate treatment behaviors in a continuous and predictive manner.

Although our study has revealed that the proposed model is effective in predicting treatment effects, even more complex analysis and evaluation tasks remain to be addressed. In this study, patient characteristics are generated using the data collected at a single time point. However, the dynamic nature of patient characteristics is often essential in the adoption of treatment interventions. In treatment processes, a patient status may be changed dynamically, and new evidence often becomes available at certain time points, which inevitably influences physician decisions on treatment selection. To address this challenge, our model should incorporate richer execution information into the learning, so as to be more intelligent in terms of treatment adoption and treatment effect improvement.

Moreover, the proposed work simply uses one treatment property, namely the treatment type, as features. This is not entirely consistent with clinical practice. In actual clinical settings, medications with different dosages and frequencies may be grouped into many treatment variants according to the physical conditions of individual patients. To address this problem, the significant potential of EHR data is required to be exploited for treatment effect estimation in a fine-grained manner.

A further limitation of our proposed model is that the causal interactions between patient status and treatments are not considered. Causal interaction analysis may be useful to identify unexpected changes in patient characteristics and explain why scheduled treatments are changed to guarantee the expected treatment effects in an interpretable manner. That is, such an approach may provide interpretable prediction on treatment effects given a specific patient status. Note that this is an open medical problem and could be addressed by mining a large amount of EHR data in a maximum-informative manner. Substantial research is still necessary to make such mining both effective and efficient.

## Conclusions

In this work, we have addressed quite a challenging problem in medical informatics, namely utilizing a large volume of observational data for TEP. We have proposed a novel model for extracting robust and discriminative representations of patient samples from their EHR data. We further improved the representation and discrimination power of the features by using adversarial loss to explore the correlational information between patient statuses and treatments. Our proposed model was evaluated on two real clinical datasets pertaining to ACS and HF, and collected from the cardiovascular department of a Chinese hospital. The experimental results demonstrate significant improvements in TEP compared to state-of-the-art methods. An interesting finding is that treatments are conditioned on patient clinical statuses and may result in varying outcomes. This inspires us to explore the correlations between patient characteristics and treatments further for promptly and accurately predicting treatment effects in our future work.

## Data Availability

The datasets used during current study are not available due to the data protection policy of our collaborative hospital.

## Abbreviations

ACEI: Angiotensin converting enzyme inhibitor
ACS: Acute coronary syndrome
ADTEP: Adversarial deep learning model for treatment effect prediction
AE: Auto-encoder
ARB: Angiotensin receptor blockers
AUC: Area Under the receiver operating characteristic Curve (AUC)
BMI: Body mass index
CABG: Coronary artery bypass grafting
CCB: Calcium channel blocker
CCR: Creatinine clearance
CK: Creatinine kinase
CKMB: Creatine kinase MB
cTnT: Cardiac troponin T
DBP: Diastolic blood pressure
DTEP: Deep learning model for treatment effect prediction
ECG: Electrocardiogram
EHR: Electronic health records
GAN: Generative adversarial network
HF: Heart failure
IPS: Inverse propensity score
LR: Logistic regression
LVEF: Left ventricular ejection fraction
MACE: Major adverse event prediction
PCI: Percutaneous coronary intervention
RCT: Randomized controlled trial
ROC: Receiver operating characteristic
SBP: Systolic blood pressure
SD: Standard deviation
SVM: Support vector machine
TEP: Treatment effect prediction
